# Rapid antigen detection of severe acute respiratory syndrome coronavirus-2 in stray cats: A cross-sectional study

**DOI:** 10.14202/vetworld.2024.1611-1618

**Published:** 2024-07-26

**Authors:** Ronaldy Santana Santos, Daniel Antônio Braga Lee, Marina dos Santos Barreto, Eloia Emanuelly Dias Silva, Pamela Chaves de Jesus, Pedro Henrique Macedo Moura, Deise Maria Rego Rodrigues Silva, Jessiane Bispo de Souza, Taynar Lima Bezerra, Patricia Oliveira Meira Santos, Adriana Gibara Guimarães, Lucas Alves da Mota Santana, Carlos Roberto Prudencio, Lysandro Pinto Borges

**Affiliations:** 1 Department of Pharmacy, Federal University of Sergipe (UFS), São Cristóvão, SE, Brazil; 2 Department of Veterinary Medicine, Federal University of Sergipe (UFS), São Cristóvão, SE, Brazil; 3 Graduate Program in Dentistry, Federal University of Sergipe (UFS), São Cristóvão, SE, Brazil; 4 Immunology Center, Adolfo Lutz Institute, São Paulo 01246-902, Brazil; 5 Interunits Graduate Program in Biotechnology, University of São Paulo, São Paulo 05508-000, Brazil; 6 Department of Immunology, Institute of Biomedical Sciences, University of São Paulo, São Paulo 05508-000, SP, Brazil

**Keywords:** animals, antigen, cats, severe acute respiratory syndrome coronavirus-2

## Abstract

**Background and Aim:**

Although reverse zoonotic transmission events from humans to domestic cats have been described, there is currently little evidence of severe acute respiratory syndrome coronavirus-2 (SARS-CoV-2) circulation in stray cats. Due to the evidence of natural and experimental infections in cats and the capacity to disseminate the virus among them, this study aimed to identify the SARS-CoV-2 antigen in stray cats from the Federal University of Sergipe in Brazil.

**Materials and Methods:**

One hundred twenty six stray cats from the university were screened for SARS-CoV-2 antigens by random sampling. Throat swab samples were tested for the virus using rapid antigen detection tests.

**Results:**

Of the 126 animals tested, 30 (23.60%) were positive for SARS-CoV-2 antigens. To our knowledge, for the first time, this study detected the SARS-CoV-2 antigen in stray cats and confirmed the presence of SARS-CoV-2 infections in Brazil’s stray cat population.

**Conclusion:**

The detection of SARS-CoV-2 in stray cats poses a risk for infected and healthy animals and possibly for humans who attend the university daily. As a limitation of the study, the small sample size necessitates caution when interpreting the results. This underscores the need for further research in this area to help control diseases in stray animals during potential pandemics. This highlights the need for monitoring and controlling the spread of the virus in stray animal populations.

## Introduction

The *Coronaviridae* family, order *Nidovirales*, includes the single-stranded RNA-enveloped viruses known as *Coronavirinae* or coronaviruses (CoVs), divided into four distinct genera [1]. Alpha- and beta-CoVs from their respective genera bring about significant respiratory, gastrointestinal, and systemic illnesses in various mammals, including humans [2]. These two genera, *Gammacoronavirus* and *Deltacoronavirus*, primarily cause respiratory and enteric infections in avian and porcine species [3]. Since the beginning of the 21^st^ century, humanity has already experienced the emergence of three new CoVs, belonging to the *Betacoronavirus* genus: Severe acute respiratory syndrome CoV (SARS-CoV) in 2003, Middle East respiratory syndrome CoV in 2012, and SARS-CoV-2 in late 2019 [4]. These epidemics arose from infectious agents crossing the species barrier from wild reservoirs such as horseshoe bats (*Rhinolophus affinis*), camels (order *Camelus*), pangolins (order *Pholidota*), and masked palm civets (*Paguma larvata*) [5–10]. The origin of SARS-CoV-2 as a zoonotic disease has yet to be definitively determined.

Domestic animals that live in close contact with humans and are infected with SARS-CoV-2, primarily cats and dogs, pose a threat to human health through potential transmission [11]. Infections in cats have been reported worldwide, especially in homes or environments where animals are in close contact with positive and quarantined humans [12]. The first case of natural transmission from humans to a cat occurred in Belgium, 1 week after its owner tested positive for SARS-CoV-2 [13]. The role of stray, feral, and shelter cats has attracted little research [14]. The infection rate of SARS-CoV-2 in stray cats is lower than in owned cats. A recent study by Sirakov *et al*. [15] demonstrated that using a Human Leukocyte Antigen – DR isotype (HLA-DR), by method enzyme-linked immunosorbent assay (ELISA) for detecting anti-SARS-CoV-2 antibodies in cats revealed a higher seroprevalence in stray cats compared to domestic cats in Bulgaria, with 83.33% positive results in stray cats versus 41.18% in domestic cats. Stray animals can acquire infectious diseases through contact with infected animals or humans and contaminated environments such as sewage and varied surfaces [14, 16–18]. A study reported natural and experimental infections in cats [19]. Both types of exposure led to subclinical and symptomatic disease in these animals [20]. Cats exhibit respiratory ailments in symptomatic diseases with coughing and sneezing, eye and nasal discharge, lethargy, anorexia, vomiting, and diarrhea [13, 21]. 70–100-day-old cats are most vulnerable to severe disease and fatalities [21]. The length of cats’ infection and viral shedding ranged from a few days to several weeks in various investigations [14].

There are three relations commonly researched with regard to SARS-CoV-2: Human-to-cat transmission, cat-to-cat transmission, and the relation of cats to other animal species. Cats, in addition to being susceptible to infections, are capable of spreading the disease among them [22]. A study found a high seroprevalence of anti-SARS-CoV-2 antibodies in stray cats, indicating significant intra-species transmission [15]. Both clinically affected and asymptomatic, Cats have been documented to disperse viral particles from their oral and nasal secretions, thereby facilitating direct or indirect transmission to other cats and even non-feline species [19, 23, 24]. While naïve cats are susceptible to contracting the disease from infected ones, SARS-CoV-2 infections induce an immune response in cats, which provides protection against re-infection [24, 25]. Domestic and farm animals primarily contract illnesses from humans [26]. While it has been highly reported that humans are responsible for infections in domestic and stray cats, the role of cats in the transmission of the disease to humans has not been confirmed [27]. Although domestic cats are more susceptible, infections have been reported in abandoned and stray animals, which can act as SARS-CoV-2 carriers and spreaders [28]. Oude Munnink *et al*. [29] showed that the virus can be transmitted between humans and animals and back to humans, as evidenced at a mink farm in the Netherlands.

In Brazil, the first case of SARS-CoV-2 in a domestic cat was reported in late 2020 [30]. Serological data suggest that not only pets owned by households with COVID-19 cases but also stray animals are being exposed to SARS-CoV-2 during the COVID-19 pandemic [31]. Studies on the occurrence of infections in stray animals are scarce but extremely important in the scope of One Health, as cats are one of the most popular pets and often live in close contact with humans [32]. Methods for detecting SARS-CoV-2 in cats include molecular techniques, such as conventional nested polymerase chain reaction (PCR) based on the SARS-CoV-2 N gene, and serological and immunoenzymatic methods, such as HLA-DR ELISAand multi-species ELISA kits [15, 33].

Therefore, it is clear that cats are potential hosts for SARS-CoV-2 infections while also being capable of disseminating the disease to healthy individuals. Identifying infections in stray animals is essential for collecting epidemiological data and implementing control programs. The aim of this study was to introduce an adaptive method for the rapid detection of SARS-CoV-2 antigens in stray cats at a Brazilian university.

## Materials and Methods

### Ethical approval

This study was approved by the Ethics Committee on the use of animals at the Federal University of Sergipe (AEC 4666041220).

### Study period and location

This cross-sectional study was conducted during October and November 2021 at the Federal University of Sergipe, located in the northeastern state of Sergipe, Brazil. The university has a total area of 192,000 m2 and approximately 300,000 members, including students, professors, and technicians (https://en.ufs.br/pagina/8167).

The cats freely roam through the university but generally live in specific groups and colonies. The university’s social projects are responsible for building structures (colonies) where the animals feed and spend the night. These are distributed throughout the university, and even with their presence, it is common to see cats in places frequented by students and university staff.

### Sampling and testing

The animals were selected with no distinction of sex, age, or physical/health condition and were captured from colonies and nearby areas. The captures and collection of secretions from the oropharyngeal mucosa of cats were carried out in the afternoon (02:00 p.m.–05:00 p.m.), a period when the cats had not yet been fed and demonstrated more interest in human presence, facilitating their capture.

After capturing and adequately containing the animals, rapid antigen detection was performed using point-of-care tests adapted for animal use. Oropharyngeal samples were collected for the detection of the SARS-CoV-2 antigen using an immunofluorescence assay (Eco F COVID-19 Ag kit with Eco Reader®, Eco Diagnostica, Brazil). This test was chosen for its high sensitivity (99.00%) and specificity (100.00%) compared to reverse transcription polymerase chain reaction (RT-PCR), based on prior validation in animals [34]. The immunofluorescence method is superior to traditional immunochromatographic tests because the reading is not visual but performed using a device that provides a semi-quantitative value. This value estimates the quantity of antigen present in the sample, which is then interpreted against a cutoff value [35].

The Eco F COVID-19 Ag® test (Eco Diagnostica Ltd, Brazil) is reported by the manufacturer to have sensitivities of 91.78% and specificities of 96.70%, with no cross-reactions observed with other CoVs, influenza, adenovirus, respiratory syncytial virus, and rhinovirus. This assay qualitatively detects the SARS-CoV-2 N protein and can detect the Omicron (B.1.1.529) variant without affecting sensitivity and specificity [36]. The biological samples were collected from throat swabs, and the results were released within 15 min, recorded in a spreadsheet, and the animals cataloged to avoid repeated collections from the same animals. The animals were observed by veterinarians for any symptoms, which were duly recorded.

### Statistical analysis and data visualization

All information (location, sex, behavior, obvious clinical condition, and rapid test result) and photographs of each cat were organized in spreadsheets. To verify a possible correlation between variables and the result of the rapid antigen detection, Fisher’s exact test and Pearson’s Chi-square test were applied using the software R version 4.1.0 (R Core Team, Vienna, Austria). We considered *a* p < 0.05 for significant results. In addition, we used the Circos® graph (Martin Krzywinski, Vancouver, Canada) (http://circos.ca/), to visualize the quantitative clinical conditions of the animals.

## Results

**Figure-1:**
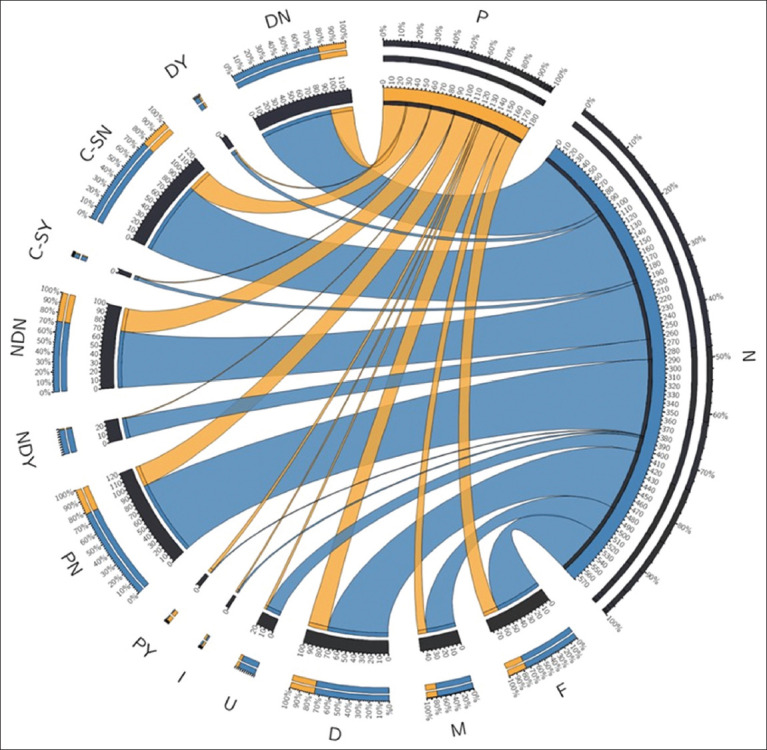
Graphical representation of the clinical conditions and quantitative results of the animals studied. The graph contains the quantitative results of the infected cats (P=Positive and N=Negative). F (P = 19; N = 59) and M (P = 11; N = 37) represent female and male sex, respectively (D=Docile; P = 25; N = 76), (U=Unsociable; P = 3; N = 18), and (I=Indifferent; P = 2; N = 2) represent feline behavior, only participants who knew the brands of vaccines they had taken. (PY=Confirmed pregnancy; P = 4; N = 1) and (PN=No pregnancy; P = 26; N = 95) represent pregnant or not pregnant females. Regarding the cats that had nasal discharge, we represented NDY (P = 1; N = 25) as cats with nasal discharge and NDN (P = 29; N=71) as cats without nasal discharge. For cats that presented with cough/sneeze, we represented them as C-SY (P = 1; N = 4) and those without Cough/Sneeze as C-SN (P = 29; N = 92). In addition, we assessed dyspnea, considering DY (P = 2; N = 5) as the cats that had dyspnea and DN (P = 28; N = 91) that did not.

**Figure-2:**
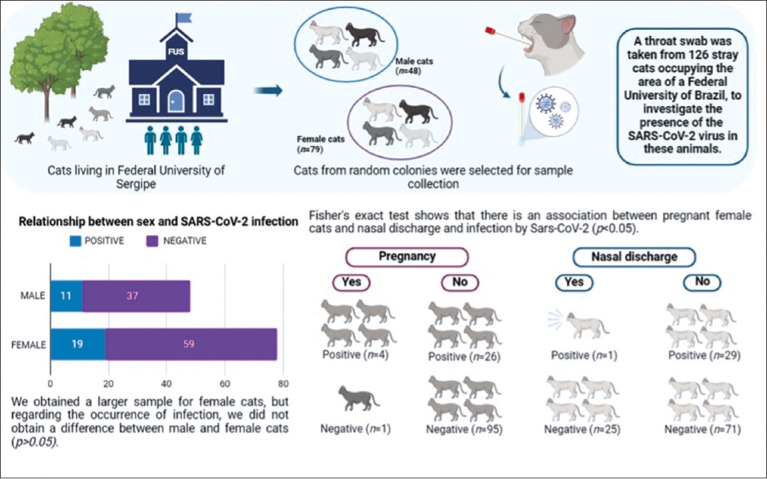
Overview of the methodology and results obtained with stray cats by the Federal University of Sergipe.

A total of 126 cats were tested for SARS-CoV-2 antigen, with 79 (62.20%) females and 48 (37.80%) males. Among them, 30 (23.60%) had a positive diagnosis, including 19 (63.30%) females and 11 (36.60%) males. It was possible to observe (through clinical observation) that a total of 26 (21.60%), 7 (5.50%), and 5 (3.90%) cats presented with nasal discharge, dyspnea, and cough and/or sneezing, respectively. In addition, through palpation, it was possible to observe that 5 (6.40%) of the analyzed females were pregnant (Figures-1 and 2, Table-1).

**Table-1:** Statistical analysis of the studied animals.

Characteristics	Positive, n (%)	Negative, n (%)	p-value	RR	CI 95%	ARR	CI 95%
	
	30 (23.80)	96 (76.20)					
Sex, n (%)							
Female	19 (63.30)	59 (61.50)	1.000^F^	1.06	0.55–2.04		
Male	11 (36.70)	37 (38.50)					
Behavior, n (%)							
Docile	25 (83.30)	76 (79.20)	0.279^Q^	0.49	0.17–1.40		
Unsociable	3 (10.00)	18 (18.80)		0.29	0.07–1.20		
Indifferent	2 (6.70)	2 (2.10)					
Pregnancy, n (%)							
Yes	4 (13.30)	1 (1.00)	0.011^F^	3.72	2.14–6.49	3.70	1.80–7.59
No	26 (86.70)	95 (99.00)					
Nasal discharge, n (%)							
Yes	1 (3.30)	25 (26.00)	0.008^F^	0.13	0.02–0.93	0.13	0.02–0.78
No	29 (96.70)	71 (74.00)					
Cough/sneeze, n (%)							
Yes	1 (3.30)	4 (4.20)	1.000^F^	0.83	0.14–4.96		
No	29 (96.70)	92 (95.80)					
Dyspnea, n (%)							
Yes	2 (6.70)	5 (5.20)	0.671^F^	1.21	0.36–4.09		
No	28 (93.30)	91 (94.80)					

n=Absolute frequency, %=Relative frequency,

F =Fisher’s exact test,

Q =Pearson’s Chi-squared test, RR=Relative risk, ARR=Adjusted relative risk, CI 95%=Confidence interval of 95%

Table-1 also represents the results of the statistical analysis that demonstrated, by means of Fisher’s exact test, an association between the presence of infections (positive test result) with pregnancy (χ^2^[1] = 9.062, p < 0.05) and nasal secretion (χ^2^[1] = 7.197, p < 0.05), considering a 95% confidence interval.

## Discussion

A total of 30/126 (23.80%) animals were tested positive for the SARS-CoV-2 antigen. Many studies have sought to report natural infections in cats worldwide with a wide variation in prevalence: 0.00% [27], 3.51% [33], and 14.70% [37]. This variation can be related to the exposure of animals to infected humans, which is directly related to the human infection rates of each country and internal measures such as quarantine and lockdowns. Even though the cats analyzed in the present study are stray, it is likely that the cases observed at the university emerged from human infection, probably from students and employees who are responsible for the health care and feeding of the cats. Humans primarily transmit SARS-CoV-2 to cats, particularly in quarantine and domestic isolation cases [26]. In docile animals, the infection ratio is higher due to their closer contact with humans compared to wild animals. Our earlier research [34] supports these findings by identifying immunoglobulin (Ig)M and IgG antibodies to SARS-CoV-2 and investigating potential cross-reactivity with feline CoVs and parvovirus. Moreover, the analysis of antigens in negative stool samples further confirmed the presence of the infection, aligning with the results of this study.

20.60% (26/126) of studied cats exhibited nasal discharge, 5.50% (7/126) presented with dyspnea, and 3.90% (5/126) coughed or sneezed, complicating treatment due to their wild behavior and restricting resources from social projects. The study revealed a positive correlation between nasal discharge in cats, pregnancy, and infections. Respiratory symptoms have been reported in numerous cat studies [14]. Given the limited number of tested animals and the small sample size of positive animals with clinical symptoms, the ratio of positive animals and nasal discharge should be interpreted with great caution. Multiple animals should undergo new testing procedures.

No studies have yet investigated the link between cat pregnancy and SARS-CoV-2 susceptibility. 5 females (6.40%) were found to be both pregnant and positive for SARS-CoV-2 antigens (Figures-1 and 2, Table-1). Older and domestic cats have lower infection rates for respiratory pathogens compared to younger and stray cats, as observed in a study in Bulgaria [15, 37]. Animals with pre-existing immunosuppression conditions are more likely to contract SARS-CoV-2 infections due to their weakened immune response. For the 1^st^ time, Villanueva-Saz *et al*. [27] documented infections in stray cats from Spain. Animals with inadequate health, housing, and food conditions, and concurrent infections with *Toxoplasma gondii*, *Leishmania infantum*, or feline immunodeficiency virus, are more susceptible to SARS-CoV-2 infections. In addition, co-infections and cross-reactivity from other pathogens presenting similar symptoms, such as *Mycoplasma*, Chlamydia, and Feline Herpesvirus, should be considered when diagnosing SARS-CoV-2 in cats [38]. The environment and climate significantly influence the transmission of the virus. According to studies [15, 39], climate conditions in different regions impact the spread of SARS-CoV-2.

Based on limited research utilizing rapid antigen detection (RAD) tests, some authors have successfully isolated viruses and identified viral RNA from the oral and/or throat swabs of infected cats [24, 25, 40, 41]. Although there is a lack of studies based on RAD tests, many authors were able to perform viral isolation and/or viral RNA identification from oral and/or throat swabs of infected cats [24, 25, 40, 41]. The detection of SARS-CoV-2 viral particles in oral and throat secretions of animals allows for the identification of infections through RAD tests. RT-PCR, while being the gold standard test for SARS-CoV-2 detection, offers advantages such as quick results, technical simplicity, and lower cost compared to other diagnostic tests [42]. RAD tests are ideal for making quick diagnostic decision in field conditions.

The RAD sensitivity and specificity for SARS-CoV-2 depend significantly on factors like brand and experimental design (https://www.who.int/publications/i/item/antigen-detection-in-the-diagnosis-of-sars-cov-2infection-using-rapid-immunoassays). The sensitivity of detecting diseases using nasal or nasopharyngeal swabs and comparing it with RT-PCR varies greatly (0.00%–94.00%) depending on disease stage, but specificity remains high (>97.00%). The World Health Organization recommends antigen-detecting rapid diagnostic tests (Ag-RDTs) with a minimum performance of ≥80.00% sensitivity and ≥97.00% specificity, per the standard set by the reference assay (https://www.who.int/publications/i/item/antigen-detection-in-the-diagnosis-of-SARS-COV-2infection-using-rapid-immunoassays). In samples with high viral loads, such as those from the initial stages of infection, rapid diagnostic tests (RDTs) are more effective.

In addition, it is important to emphasize that the *Coronaviridae* family has many genera that affect a wide variety of animals, including the feline enteric coronavirus (FECV) and the feline infectious peritonitis coronavirus (FIPV), both of which are responsible for acute diseases in cats [43]. No serological reaction has been observed between FECV/FIPV and SARS-CoV-2 [27, 28, 44]. Cross-reactivity is not an issue in the context of antigen detection using the RAD test, as it targets unique SARS-CoV-2 nucleoproteins.

Several outbreaks of SARS-CoV-2 infection were marked by numerous positive animal colonies. Bosco-Lauth *et al*. [24] experimentally infected cats and confirmed direct transmission between them. The study used viral isolation and qRT-PCR to confirm that animals in experimentally and direct contact with infected groups shed viral particles in their oral and nasal secretions for 5–7 days following infection. Shi *et al*. [22] demonstrated the susceptibility of cats to SARS-CoV-2 infections and reinforced the transmission between animals by respiratory droplets. On the other hand, many colonies had no cases of cats infected by SARS-CoV-2.

This result can be explained by the distribution of animals across the university. Even though cats are stray and can freely roam through the campus, they tend to be close together or in specific colonies, limiting the spread of disease among them. Cats can develop antibodies against SARS-CoV-2, which protect them from reinfections following either natural or experimental infections [43, 45].

## Conclusion

Based on RDA test results, this study confirms that stray cats from the Federal University of Sergipe have been infected with SARS-CoV-2. These findings underscore the infection’s occurrence in stray animals and heighten concerns about its prevalence. Although the study has limitations, such as the small sample size and the uneven number of cats with respect to their characteristics and conditions, these issues underscore the need for caution in interpreting the results. These limitations also highlight the importance of conducting further research with larger, more uniformly categorized samples. The detection of SARS-CoV-2 in stray cats emphasizes the critical need for ongoing research and monitoring, including studies with larger sample sizes, more uniform categorization of animal conditions, and the use of diverse diagnostic methods. Additionally, it is important to investigate the transmission dynamics of SARS-CoV-2 across different animal populations and their interactions with humans to better understand and manage the virus’s spread.

## Data Availability

The supplementary data can be made available by the corresponding author upon request.

## Authors’ Contributions

RSS, DABL, TLB, and POMS: Conceived and designed the study. EEDS, PCJ, JBS, RSS, MSB, PHMM, DMRRS, and CRP: Performed the study. DABL, EEDS, PCJ, PHMM, DMRRS, RSS, MSB, and LPB: Analyzed and interpreted the data. DABL, RSS, LPB, and CRP: Performed statistical analysis. DABL, RSS, LPB, and CRP: Wrote the manuscript. RSS, MSB, AGG, LAMS, CRP, and LPB: Revised the manuscript. AGG, LAMS, CRP, and LPB: Supervised the study. LPB: Project administration. All authors have read, reviewed, and approved the final manuscript.
